# Experience with PEEK Clamp Fixation of Custom Porous Hydroxyapatite Implants in Cranioplasty: A Preliminary Single-Center Feasibility Case Series

**DOI:** 10.3390/jcm15187152

**Published:** 2026-09-15

**Authors:** Alberto Vandenbulcke, Nathalie Zimmermann, Rodolfo Maduri

**Affiliations:** 1 Department of Clinical Neurosciences, Service of Neurosurgery, Lausanne University Hospital (CHUV), 1011 Lausanne, Switzerland; 2 Department of Orthopedics and Traumatology, Spine Surgery, Hôpital Riviera Chablais, 1847 Rennaz, Switzerland

**Keywords:** cranioplasty, fixation system, porous hydroxyapatite implant, case series

## Abstract

**Background**: Cranioplasty using porous hydroxyapatite (PHA) implants is an established technique for cranial reconstruction due to its favorable osteointegration properties. However, the intrinsic fragility of PHA implants before complete osteointegration may limit the use of conventional rigid fixation systems. Although non-absorbable sutures remain the manufacturer-recommended fixation method, alternative fixation strategies have been proposed to improve implant stability during the early postoperative period. **Methods**: This retrospective single-center case series describes our preliminary clinical experience with a polyether-ether-ketone (PEEK) clamp fixation system (Cranial LOOP^®^) used in combination with customized porous hydroxyapatite implants. Seven consecutive patients undergoing cranioplasty between January and December 2019 were included. Clinical follow-up, postoperative imaging, implant stability, fixation-related complications, and cosmetic outcomes were retrospectively reviewed over a median follow-up period of 48 months. **Results**: All implants were satisfactorily positioned on immediate postoperative CT imaging, and no fixation-related displacement or implant fracture was observed during follow-up. No patient required revision surgery because of fixation failure. Radiological follow-up demonstrated progressive bone integration at the implant margins in 6/7 patients (85.7%). Cosmetic outcomes were generally satisfactory according to clinical assessment, although interpretation of these findings is limited by the subjective evaluation method and the absence of blinded assessment. **Conclusions**: This preliminary retrospective case series suggests that fixation of customized porous hydroxyapatite implants using Cranial LOOP^®^ is technically feasible. Owing to the small sample size, retrospective design, lack of a control group, and subjective outcome assessment, no conclusions regarding comparative safety, mechanical superiority, or clinical effectiveness can be drawn. Larger prospective comparative studies are required to further evaluate this fixation strategy.

## 1. Introduction

Cranioplasty is a well-established neurosurgical procedure performed to restore cranial defects following decompressive craniectomy, trauma, infection, tumor surgery, or congenital abnormalities [1]. Besides protecting the underlying brain, cranioplasty restores cranial contour and may improve neurological function and quality of life.

Patient-specific porous hydroxyapatite (PHA) implants have become an increasingly common reconstructive option because of their excellent biocompatibility, osteoconductive properties, and potential for progressive osseointegration [2]. Nevertheless, until biological integration occurs, PHA implants remain relatively fragile and may be susceptible to fracture or displacement during implantation or the early postoperative period [2,3].

Unlike titanium or PEEK implants, porous hydroxyapatite prostheses may be susceptible to mechanical damage when rigid fixation requiring drilling or screw placement through the implant is used [4,5,6]. The manufacturer therefore recommends fixation using non-absorbable sutures. At our institution, a polyether-ether-ketone (PEEK) clamp fixation system (Cranial LOOP^®^) was adopted as an alternative fixation strategy based on surgeon preference for a circumferential fixation method allowing intraoperative adjustment without drilling or screw placement through the PHA implant. Its adoption was not prompted by a documented institutional failure rate with suture fixation. Rather, it represented a technical choice intended to provide stable fixation while limiting direct mechanical manipulation of the prosthesis. Whether this approach provides any clinical or mechanical advantage over conventional suture fixation remains unknown [7].

The objective of the present study is to describe the initial single-center experience and evaluate the technical feasibility of combining Cranial LOOP^®^ fixation with customized porous hydroxyapatite implants during cranioplasty.

## 2. Materials and Methods

### 2.1. Study Design

This retrospective single-center observational case series describes our initial clinical experience with the use Cranial LOOP^®^ (NEOS Surgery S.L., Barcelona, Spain) for fixation of customized PHA cranial implants.

The study was conducted at the Department of Clinical Neurosciences, Service of Neurosurgery, Lausanne University Hospital (CHUV), Switzerland. All consecutive patients undergoing cranioplasty with a customized PHA implant secured using the Cranial LOOP^®^ between January and December 2019 were retrospectively identified from our institutional surgical database.

The primary objective was to evaluate the technical feasibility of this fixation technique. No comparative analysis with conventional fixation methods was planned because no control group was available.

The study protocol was approved by the Cantonal Ethics Committee (CER-VD, Project ID 2024-02281), and all procedures were conducted in accordance with the Declaration of Helsinki.

### 2.2. Patient Selection

Inclusion criteria

Patients were eligible if they fulfilled the following criteria:

- age ≥ 3 years;
- cranial defect reconstructed using a customized PHA implant (CustomBone^®^, Fin-Ceramica S.p.A, Faenza, Italy);
- fixation performed exclusively using the Cranial LOOP^®^ system;
- postoperative CT imaging available;
- minimum clinical follow-up of 6 months.

Exclusion criteria

Patients were excluded if they:

- underwent cranioplasty using another implant material;
- received another fixation technique;
- had incomplete clinical documentation;
- had no postoperative imaging available.

All eligible patients treated during the study period were included consecutively, minimizing selection bias. All consecutive patients in whom a customized PHA implant was fixed exclusively with the Cranial LOOP^®^ system. The surgical procedures were performed as part of routine clinical care, without prospective study-specific data collection. The present retrospective study was conceived in 2023. Due to the retrospective nature of the study, the general contentment form for research was applicable according to the competent Ethical Board (CER-VED).

No study-specific data were collected, extracted, or analyzed before ethical approval, and retrospective data collection commenced only after approval was granted in January 2025.

In patients undergoing delayed cranioplasty after infection, reconstruction was performed only after complete clinical resolution of infection, completion of systemic antibiotic therapy, and multidisciplinary agreement between neurosurgeons and infectious disease specialists.

The interval between infection resolution and cranioplasty varied according to the clinical scenario and was individualized for each patient.

### 2.3. Study Outcomes

The primary endpoint was technical feasibility of Cranial LOOP^®^ fixation.

Technical feasibility was defined as successful implantation with stable intraoperative fixation and satisfactory implant position on immediate postoperative CT.

The following secondary outcomes were evaluated.

- Implant displacement: on serial CT imaging during follow-up examinations. Implant displacement was reported as present/absent;
- Radiological integration: assessed qualitatively on serial CT examinations and defined as progressive continuity between the implant margins and native skull, absence of enlarging radiolucent gaps at the implant–host interface, and evidence of marginal bone remodeling. Because imaging was obtained as part of routine clinical follow-up and was not evaluated using a validated quantitative method or blinded independent review, these findings are reported as descriptive evidence of radiological integration rather than definitive measurements of osseointegration.
- Hardware-related complications: intraoperative or postoperative adverse events potentially related to the implant or fixation system, including device malfunction or breakage, implant fracture or displacement, clinically evident implant mobility, wound dehiscence, surgical-site infection, cerebrospinal fluid leakage, symptomatic postoperative hematoma, foreign-body reaction, and revision surgery.
- Cosmetic outcome assessed using the Global Aesthetic Improvement Scale (GAIS), a five-point ordinal scale comparing the postoperative appearance with the pretreatment condition. The scale ranges from 1 (very much improved) to 5 (worse), with lower scores indicating better aesthetic outcomes [8]. The assessment included cranial symmetry, contour restoration, skin adaptation and implant palpability. Because of the retrospective nature of the study, the cosmetic assessment was performed by the treating surgeon during routine outpatient follow-up and was not independently or blindly evaluated.

### 2.4. Follow-Up

According to the institutional protocol, patients underwent immediate postoperative CT approximately 48 h after surgery, with follow-up CT examinations at approximately 6 and 12 months and whenever clinically indicated. Additional imaging was performed according to the underlying pathology.

Clinical assessment included wound healing, implant stability, neurological examination, evidence of infection, and the need for revision surgery.

One patient relocated abroad after the six-month visit and was therefore unavailable for further follow-up.

### 2.5. Device Description

Cranial LOOP^®^ is designed to secure cranial bone flaps following craniotomy. It consists of a lower platform with cable ties, an upper platform with toothed slots, a grip handle for controlled tightening, and an applier ensuring safe and repeatable closure. The device, made of biocompatible PEEK OPTIMA™ (Invibio Biomaterials Solutions, Thornton-Cleveleys, UK), does not require specialized instrumentation for placement or removal.

The implantation process involves inserting the lower platform in the epidural space, placing the bone flap between the platforms, and securing it by pulling the handle while guiding the upper platform with the applier. Tension should not exceed 40 N to prevent structural failure. Stability is confirmed by testing for movement after fixation. Removal involves lifting the upper platform with forceps and ensuring no fragments remain in the surgical site.

Contraindications include infection, bone disease, lack of adequate tissue coverage, sinus penetration, and intolerance to PEEK. The manufacturer recommends using at least three Cranial LOOP^®^ per craniotomy for optimal fixation. Being non-ferromagnetic, Cranial LOOP^®^ does not interfere with MRI imaging. System integrity should be monitored postoperatively, with replacement as needed.

### 2.6. PHA Prosthesis Fixation: Surgical Procedure

Patients received perioperative antibiotic prophylaxis according to institutional neurosurgical protocols.

Patients undergoing delayed cranioplasty following previous infection completed culture-directed antimicrobial treatment before reconstruction.

No patient presented clinical or laboratory evidence of active infection at the time of cranioplasty.

For the fixation of the PHA prosthesis, at least three Cranial LOOP^®^ devices are employed to ensure stable and secure positioning within the cranial defect, while larger cranial defects required four fixation devices to obtain satisfactory circumferential stability.

**Figure 1:**
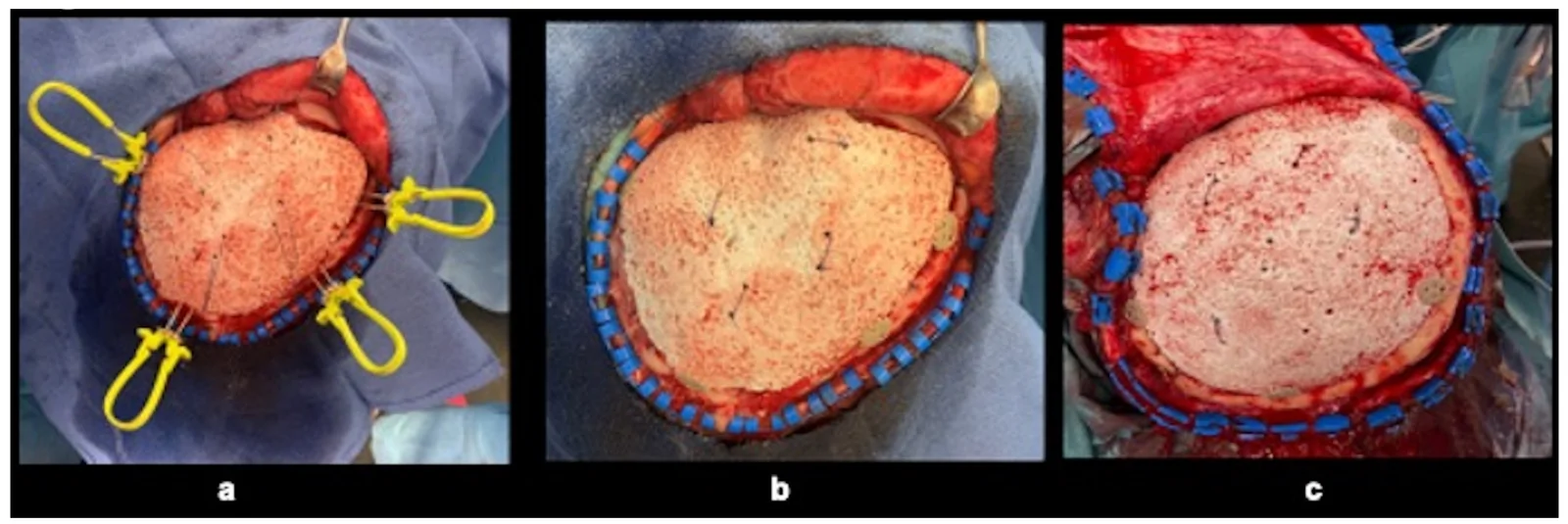
Intra-operative images show the prosthesis implantation: (**a**) Cranial LOOP^®^ devices (4) are around the prosthesis; (**b**,**c**) The Cranial LOOP^®^ are clamped and the cable ties removed, the positioned external platform shows its low profile.

The fixation procedure begins with the careful placement of the lower platform of each Cranial LOOP^®^ into the epidural space beneath the craniotomy margin, ensuring minimal manipulation to avoid dural injury. Once the prosthesis is positioned within the cranial defect, the upper platform is placed epicranially and secured by gradually applying tension using the applier (Figure 1). The double-locking mechanism of the Cranial LOOP^®^ prevents loosening and maintains stable fixation throughout the postoperative healing period.

The cable ties are then trimmed using surgical scissors, taking advantage of the device’s self-cutting feature to ensure a clean and flush fit. To confirm adequate fixation, gentle pressure is applied to the prosthesis to assess resistance to displacement. The circumferential distribution of the Cranial LOOP^®^ devices permits intraoperative adjustment of fixation points without requiring predefined fixation slots in the PHA.

The Cranial LOOP^®^ system provides a reliable and adjustable method for securing CustomBone^®^ in cranioplasty, ensuring fixation while preserving flexibility for intraoperative modifications. Its non-ferromagnetic nature allows for safe postoperative imaging, and its design minimizes the need for supplementary fixation hardware.

### 2.7. Case Example

**Figure 2:**
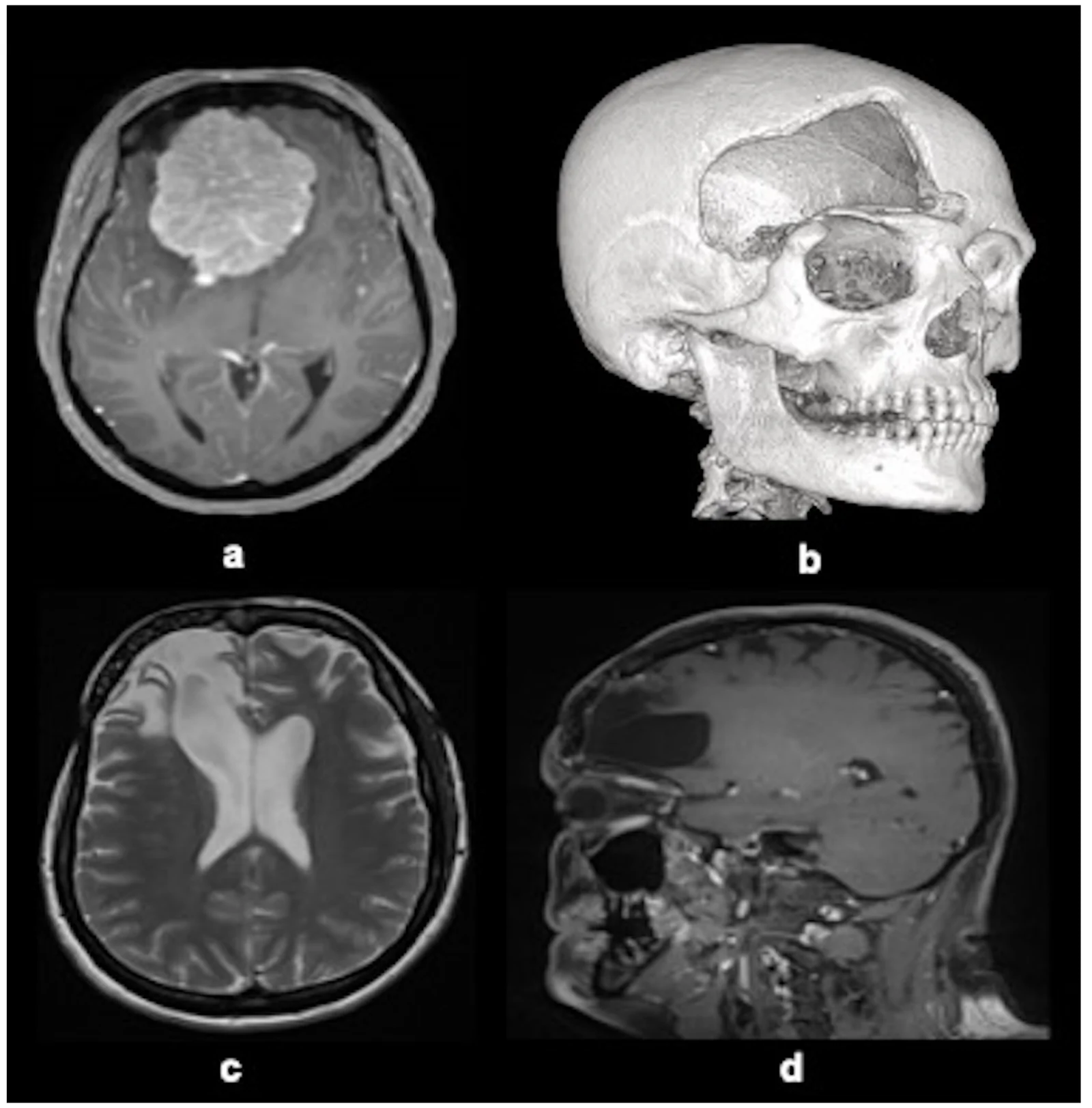
Case example MR images. (**a**) preoperative axial T1-weighted with gadolinium showing a large olfactory groove meningioma. (**b**) postoperative CT scan with 3D reconstruction showing a large aesthetic defect in the right frontal region. (**c**) Postoperative axial T2 weighted and (**d**) T1 weighted sagittal images with gadolinium showing the prosthesis fitting the cranial defect without imaging artifact.

**Figure 3:**
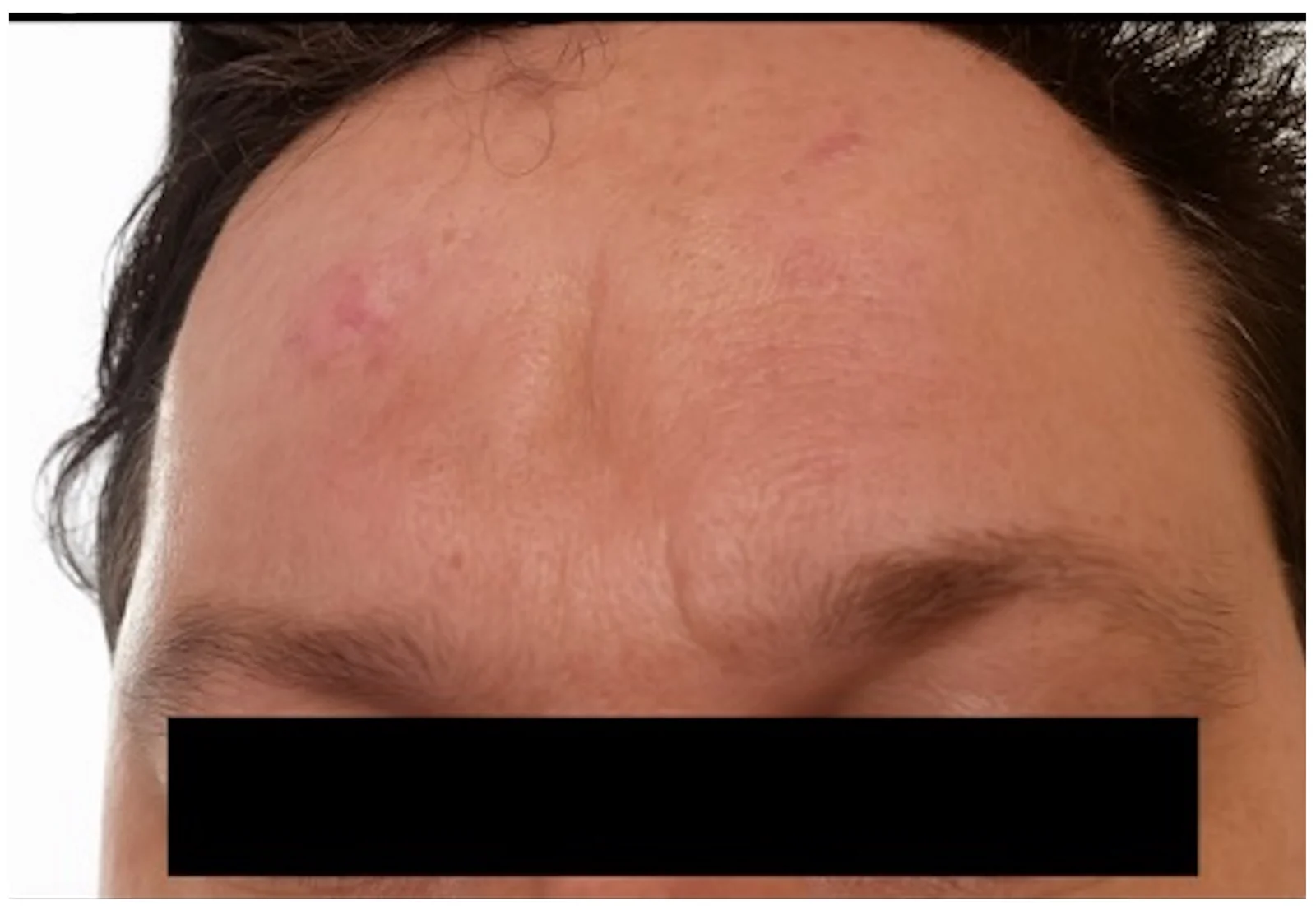
Case example: Four-years postoperative photograph showing a satisfactory aesthetic result in the right frontal region.

Patient 1 was a 51-year-old female who underwent surgery for a large olfactory groove meningioma using a right frontotemporal craniotomy (Figure 2). One month later, she underwent reoperation following postoperative infection (frontal sinus cranialization and bone flap removal). Intraoperative samples were positive for *S. epidermidis*, and she received appropriate antibiotic treatment for 6 weeks. Due to the location of the bone flap, a custom-made prosthesis was proposed to the patient to achieve the best aesthetic result. Once the treatment was completed, a fine-cut CT scan was performed to plan the 3D custom bone prosthesis, which was implanted 2 months after the end of the treatment. The aesthetic result was satisfactory, with no visible bony gap and good frontal symmetry. Long-term radiological follow-up was performed due to the risk of meningioma recurrence. At 4 years, no signs of fracture or displacement were observed, and a satisfactory aesthetic result was reported (Figure 3).

### 2.8. Use of Artificial Intelligence-Assisted Tools

During the preparation and revision of this manuscript, the authors used ChatGPT (OpenAI, Version 26.908.40834) solely for English-language editing, grammatical correction, and improvement of the clarity and readability of the text. The AI tool was not used for study design, data collection, data generation, data analysis, statistical analysis, interpretation of the results, or formulation of scientific conclusions. All AI-assisted text was critically reviewed and edited by the authors, who take full responsibility for the accuracy, integrity, and final content of the manuscript.

## 3. Results

### 3.1. Population (Table 1)

Seven consecutive patients met the inclusion criteria and underwent cranioplasty using a customized porous hydroxyapatite implant fixed with the Cranial LOOP^®^ between January and December 2019.

The mean patient age was 38.8 years (range 3–61 years). Four patients were male (57%) and three were female (43%). Trauma represented the most frequent initial pathology (5/7 patients, 71%), while infection following previous cranioplasty was the most common indication for reconstruction (5/7 patients).

**Table 1:** Characteristics of the population.

Patient Number	Age	Sex	Initial Pathology	Indication for Cranioplasty	Defect Location
1	51	F	Cerebral tumor	Infection	Frontal
2	52	M	Trauma	Infection	Fronto-parieto-temporal
3	56	M	Stroke	Infection	Fronto-parieto-temporal
4	39	F	Trauma	Other prosthesis displacement	Frontal
5	61	M	Trauma	Infection	Fronto-parieto-temporal
6	3	F	Trauma	Infection	Fronto-parieto-temporal
7	10	M	Trauma	Osteolysis	Fronto-parieto-temporal

Legend. F: female, M: male.

### 3.2. Early Postoperative Outcomes

Results are reported in Table 2. Cranioplasty was successfully completed in all seven patients without intraoperative device failure or technical difficulties requiring conversion to another fixation technique.

A minimum of three Cranial LOOP^®^ fixation devices was used in each patient. Four or five fixation devices were used for larger cranial defects according to intraoperative judgment.

No intraoperative prosthesis fractures occurred during implantation.

**Figure 4:**
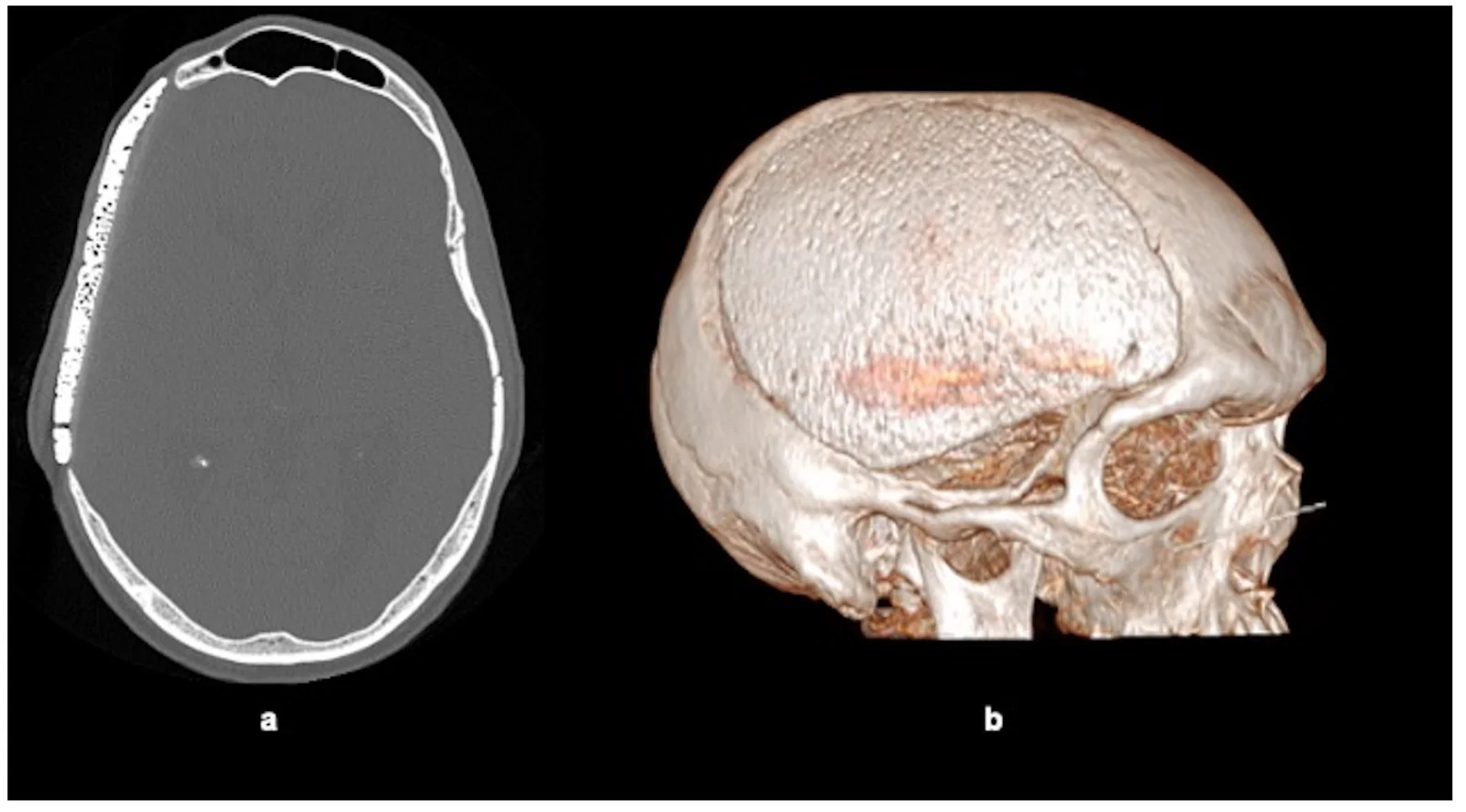
Bone CT-scan 48 h post-operatively: (**a**) axial bone CT image showing satisfactory placement of the cranioplasty flap; (**b**) 3D reconstruction of the CT scan.

Postoperative CT imaging performed approximately 48 h after surgery (Figure 4) demonstrated satisfactory positioning of all implants.

No patient demonstrated:

- implant displacement;
- epidural hematoma requiring surgery;
- cerebrospinal fluid leakage;
- fixation device failure.

No early wound complications were observed.

### 3.3. Clinical and Radiological Follow-Up Outcomes 

The median clinical follow-up was 48 months (range 6–60 months). One patient (14.7%) completed the scheduled 6-month follow-up but subsequently relocated abroad and was therefore unavailable for long-term evaluation.

During follow-up, no patient underwent revision surgery because of implant instability or fixation failure. No postoperative surgical-site infections were observed during follow-up.

No fixation failure nor foreign-body reactions were observed.

**Figure 5:**
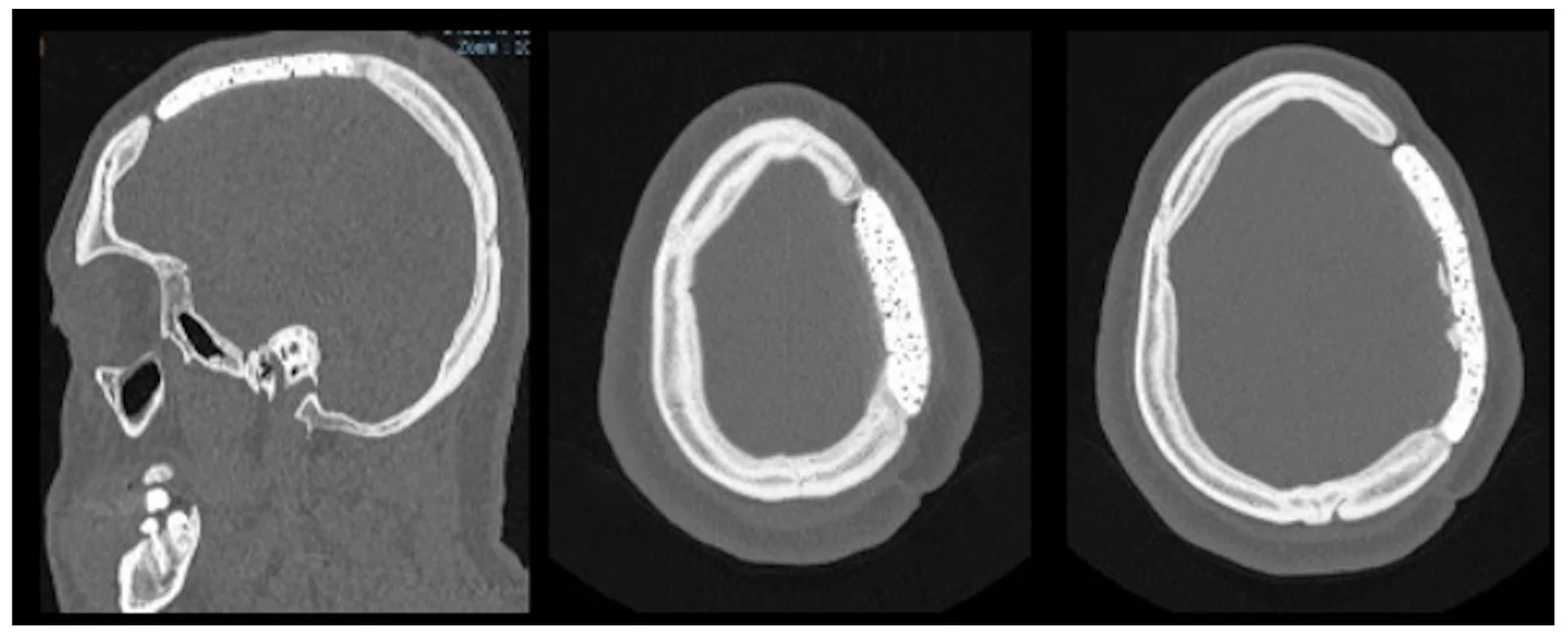
Cerebral CT scan at 6-month follow-up demonstrating radiological continuity and bone remodeling at the prosthesis margins, findings consistent with progressive radiological integration.

Radiological integration was evaluated by within-patient comparison of the immediate postoperative CT examination with the available follow-up CT examinations. Follow-up CT imaging was available at 12 months in six patients (6/7), whereas Patient 7 had radiological follow-up limited to 6 months (Figure 5).

Clinical follow-up duration was considered separately from radiological follow-up and is reported independently in Table 2. Radiological integration and absence of prosthesis displacement were assessed qualitatively by within-patient comparison of matched serial CT examinations. Findings consistent with progressive integration were defined as increasing continuity at the implant–bone interface, absence of an enlarging radiolucent gap, and evidence of marginal bone remodeling. Because imaging assessment was qualitative, non-blinded, and performed without validated quantitative measurements, the findings were interpreted descriptively radiological changes consistent with progressive integration rather than definitive evidence of osseointegration.

Cosmetic outcomes were assessed by the treating surgeon during routine postoperative outpatient follow-up using the Global Aesthetic Improvement Scale (GAIS). The assessment was based on comparison with the preoperative appearance and included cranial symmetry, contour restoration, skin adaptation, and implant palpability. As the GAIS has not been specifically validated for cranioplasty and the evaluation was neither blinded nor independent and did not include a patient-reported measure, the resulting scores were considered subjective and exploratory.

## 4. Discussion

The present study describes our initial institutional experience using a PEEK clamp fixation system, Cranial LOOP^®^, used in combination with customized porous hydroxyapatite implants for cranioplasty. Rather than evaluating superiority over conventional fixation methods, the purpose of this study was to describe the technical feasibility of this approach and report the associated clinical observations at follow-up.

Porous hydroxyapatite has become an increasingly attractive material for cranial reconstruction because of its osteoconductive properties and ability to integrate progressively with native bone [2,9]. Nevertheless, the relatively low mechanical resistance of porous hydroxyapatite before complete osseointegration remains an important material-related limitation, particularly during implantation and the early postoperative period [3,4,10]. This limitation should not be interpreted as being specifically attributable to suture fixation. Published series of PHA cranioplasty have reported implant fracture and displacement or mobilization, although most studies do not provide fixation-specific complication rates. In a multicenter adult series of 494 patients, fracture and implant mobilization occurred in 1.01% and 0.4% of patients, respectively [8]. In a multicenter pediatric series, fracture and displacement were reported in 6.2% and 4.6%, respectively [10]. The latter authors noted that PHA implants are commonly secured using sutures through prefabricated holes and proposed that this fixation method may contribute to implant mobility; however, no controlled comparison with alternative fixation systems was performed [10]. Therefore, the available evidence does not establish a specific failure rate attributable to non-absorbable suture fixation.

In our institution, the use of Cranial LOOP^®^ was therefore not introduced in response to an observed excess of complications with suture fixation, but as a surgeon-selected technical alternative permitting circumferential fixation without drilling or screw placement through the PHA prosthesis. The present study was designed to describe the feasibility of this approach and cannot determine whether it provides greater mechanical stability or fewer complications than conventional sutures.

In this series, fixation using the Cranial LOOP^®^ system was successfully completed in all seven patients without the need for intraoperative conversion to another fixation technique. These observations support the technical feasibility of the procedure in the selected cases studied.

Importantly, the absence of fixation-related complications in seven patients should not be interpreted as evidence of device safety. Rare complications cannot be excluded in a cohort of this size, and substantially larger prospective studies would be required to establish the safety profile of this fixation strategy.

Likewise, although progressive bone integration was observed radiologically during follow-up, radiological assessment was qualitative and based on routine clinical imaging rather than standardized quantitative measurements. Therefore, no definitive conclusions regarding osseointegration can be drawn.

Several theoretical advantages of PEEK fixation systems have been proposed in previous publications, including reduced imaging artifacts, improved implant handling, low device palpability, and favorable biomechanical characteristics [5,6,11,12]. However, these parameters were not directly evaluated in the present study. Consequently, the current results should not be interpreted as demonstrating superiority over titanium fixation systems or non-absorbable sutures.

Cosmetic outcomes should likewise be interpreted cautiously. Although postoperative appearance was generally considered satisfactory during routine clinical follow-up, aesthetic assessment was subjective, performed by the operating surgeon, and was neither blinded nor independently validated. The Global Aesthetic Improvement Scale was used descriptively during routine clinical follow-up. This scale has not been validated specifically for cranioplasty, and the assessments were performed by the treating surgeon without blinded independent evaluation or a validated patient-reported outcome measure. Consequently, the GAIS results should be regarded solely as subjective and exploratory observations and not as evidence of validated cosmetic effectiveness. A cranioplasty-specific assessment instrument, such as the validated Rostock Functional and Cosmetic Cranioplasty Score, should be considered in future prospective studies [13].

The present study has several important limitations. First, the cohort included only seven patients, limiting statistical inference and external validity. Second, the retrospective single-center design introduces potential selection and observer bias. Third, the absence of a control group precludes comparison with alternative fixation techniques. Fourth, radiological and cosmetic outcomes were assessed qualitatively without blinded review. Finally, one patient was lost to long-term follow-up after the six-month assessment.

Furthermore, operative time, fixation strength, mechanical performance, costs, and comparative safety were not evaluated. Accordingly, no conclusions regarding superiority, efficiency, safety, or cost-effectiveness can be drawn.

Despite these limitations, the present report contributes additional clinical experience regarding the use of Cranial LOOP^®^ with porous hydroxyapatite implants, a combination for which only limited clinical evidence currently exists.

Future prospective multicenter studies comparing Cranial LOOP^®^ fixation with conventional suture and titanium fixation systems are required before conclusions can be drawn regarding comparative safety, effectiveness, mechanical performance, or cost-effectiveness.

## 5. Conclusions

This retrospective case series describes the technical feasibility of using Cranial LOOP^®^ to fix customized porous hydroxyapatite cranioplasty implants. Fixation was successfully completed in all seven selected patients, with satisfactory implant positioning on immediate postoperative imaging. Given the small sample, retrospective design, and absence of a control group or objective mechanical and operative measurements, these findings should be interpreted solely as preliminary evidence of technical feasibility. No conclusions regarding superiority over sutures or other fixation methods, reduced operative time, comparative safety, mechanical performance, or cost-effectiveness can be made.

Prospective multicenter comparative studies involving larger patient cohorts are required to establish the long-term clinical performance of this fixation strategy.

## Figures and Tables

**Table 2:** Outcomes.

Patient	Cranial LOOP Devices, n	Technical Failure	Immediate CT Position	Postoperative Complication	CT Follow-Up (months)	Radiological Integration	Surgical Revision	F-U Duration (months)	GAIS Score at F-U
1	4	No	Satisfactory	None	12	Progressive	No	48	3
2	4	No	Satisfactory	None	12	Progressive	No	60	3
3	4	No	Satisfactory	None	12	Progressive	No	36	3
4	3	No	Satisfactory	None	12	Progressive	No	60	2
5	4	No	Satisfactory	None	12	Progressive	No	24	3
6	3	No	Satisfactory	None	12	Progressive	No	60	3
7	4	No	Satisfactory	None	6	NA	No	6	1

Legend: CT, computed tomography; F-U: follow-up; GAIS, Global Aesthetic Improvement Scale; NA, not assessable due to short-term follow-up.

## Data Availability

Data are not available for patient privacy.
